# Label‐free atherosclerosis diagnosis through a blood drop of apolipoprotein E knockout mouse model using surface‐enhanced Raman spectroscopy validated by machine learning algorithm

**DOI:** 10.1002/btm2.10529

**Published:** 2023-05-03

**Authors:** Sanghwa Lee, Miyeon Jue, Minju Cho, Kwanhee Lee, Bjorn Paulson, Hanjoong Jo, Joon Seon Song, Soo‐Jin Kang, Jun Ki Kim

**Affiliations:** ^1^ Biomedical Engineering Research Center Asan Medical Center Seoul Republic of Korea; ^2^ Wallace H. Coulter Department of Biomedical Engineering Emory University and Georgia Institute of Technology Atlanta Georgia USA; ^3^ Department of Pathology University of Ulsan College of Medicine, Asan Medical Center Seoul Republic of Korea; ^4^ Department of Cardiology University of Ulsan College of Medicine, Asan Medical Center Seoul Republic of Korea; ^5^ Department of Biomedical Engineering University of Ulsan, College of Medicine Seoul Republic of Korea

**Keywords:** ApoE KO mouse, atherosclerosis, nano‐sized biomarker, principal component analysis, surface‐enhanced Raman spectroscopy

## Abstract

The direct preventative detection of flow‐induced atherosclerosis remains a significant challenge, impeding the development of early treatments and prevention measures. This study proposes a method for diagnosing atherosclerosis in the carotid artery using nanometer biomarker measurements through surface‐enhanced Raman spectroscopy (SERS) from single‐drop blood samples. Atherosclerotic acceleration is induced in apolipoprotein E knockout mice which underwent a partial carotid ligation and were fed a high‐fat diet to rapidly induce disturbed flow‐induced atherosclerosis in the left common carotid artery while using the unligated, contralateral right carotid artery as control. The progressive atherosclerosis development of the left carotid artery was verified by micro‐magnetic resonance imaging (micro‐MRI) and histology in comparison to the right carotid artery. Single‐drop blood samples are deposited on chips of gold‐coated ZnO nanorods grown on silicon wafers that filter the nanometer markers and provide strong SERS signals. A diagnostic classifier was established based on principal component analysis (PCA), which separates the resultant spectra into the atherosclerotic and control groups. Scoring based on the principal components enabled the classification of samples into control, mild, and severe atherosclerotic disease. The PCA‐based analysis was validated against an independent test sample and compared against the PCA‐PLS‐DA machine learning algorithm which is known for applicability to Raman diagnosis. The accuracy of the PCA modification‐based diagnostic criteria was 94.5%, and that of the machine learning algorithm 97.5%. Using a mouse model, this study demonstrates that diagnosing and classifying the severity of atherosclerosis is possible using a single blood drop, SERS technology, and machine learning algorithm, indicating the detectability of biomarkers and vascular factors in the blood which correlate with the early stages of atherosclerosis development.

**Abbreviations**
ApoE: apolipoprotein E
ASCVD: atherosclerotic cardiovascular disease
AUC: area under the curve
CK‐MB: creatine kinase‐MB
DA: discriminant(s) analysis
DAPI: 4′,6‐diamidino‐2‐phenylindole
d‐flow: disturbed blood flow
DI: deionized
ECA: external carotid artery
EEM: external elastic membrane
H&E: hematoxylin/eosin
HMTA: hexamethylenetetramine
ICA: internal carotid artery
IF: immunofluorescence
KO: knockout
LCA: left carotid artery
LDL: low‐density lipoprotein
MR: magnetic resonance
MRI: magnetic resonance imaging
MRI‐TOF: time‐of‐flight magnetic resonance imaging
NA: numerical aperture
OA: occipital artery
PC: principal component
PCA: principal component analysis
PLS: partial least squares regression
RCA: right carotid artery
RF: radio frequency
ROC: receiver operating characteristic
SEM: scanning electron microscope
SERS: surface‐enhanced Raman spectroscopy
TOF: time‐of‐flight
WD: working distance

## 1 INTRODUCTION

Atherosclerosis is a chronic inflammatory vascular disease that is initiated by the accumulation, oxidation, and glycation of low‐density lipoprotein (LDL) cholesterol in the endothelium and progresses with the expression of pro‐inflammatory adhesion molecules and release of chemo‐attractants.^1^, ^2^ Most clinical atherosclerotic cardiovascular diseases (ASCVDs) are characterized by stable or unstable coronary disease, ischemic stroke, and peripheral arterial disease; ASCVD is the final step of a vascular inflammatory process.^3^, ^4^ Advanced atherosclerosis induces chronic luminal narrowing and ischemia of peripheral tissues.^5^ Moreover, unstable plaques with a high content of necrotic core and thin fibrous cap are prone to rupture, which may cause acute atherothrombotic events and tissue necrosis.^6^

Unfortunately, the early identification of vulnerable atherosclerotic lesions using serum biomarkers remains challenging.^2^, ^7^ Traditional risk factors such as old age, hypertension, smoking, obesity, and diabetes show a considerably low specificity in ASCVD detection. Although hyperlipidemia is related to the initiation and progression of atherosclerosis, no direct correlation has been established between the serum level of LDL cholesterol and lesion severity. The elevated serum level of C‐reactive protein, an inflammatory mediator, is associated with the presence of ASCVD and occurrence of ischemic events. However, the role of C‐reactive protein in inflammatory mechanisms and its causality in atherosclerosis has not yet been fully elucidated.^8^ Moreover, the level of C‐reactive protein poorly reflects the degree of stenosis and morphological plaque characteristics.^9^ Although cardiac troponin and creatine kinase‐MB (CK‐MB) are the key markers for the diagnosis of acute myocardial infarction, these post‐event biomarkers cannot detect stable coronary artery disease or reduce cardiovascular risks.^10^, ^11^

While angiography is the gold standard tool used to assess the severity of stenosis, the contrast luminography cannot provide information on plaque morphology or arterial wall pathology. With a high resolution, intravascular ultrasound is useful in quantifying atheroma and characterizing tissue in the catheterization laboratory. However, the extensive use of intravascular imaging in the real world is restricted by its invasiveness and high cost.^12^, ^13^

Biological samples for liquid biopsy can be derived from any secretion containing metabolism byproducts, such as tears, blood, urine, and saliva. Biomarkers and reagents present in these materials can provide a basis for diagnosis, enabling an early measurement of drug treatment outcomes.^7^, ^14^, ^15^ Biomarkers can be grouped into cells (approx. size of tens of μm), red blood cells (approx. 8 μm), bacteria (approx. 1 μm), viruses (approx. 400 nm), exosomes (several nm to tens of nm), and proteins, among others, with each group having a typical size range. The type of biomarker most appropriate for diagnosis depends upon the disease and its occurrence mechanism. In general, smaller target marker molecules in liquid samples can be detected from smaller sample quantities; however, a higher sensitivity detection technology is required.

Surface‐enhanced Raman spectroscopy (SERS) is a useful candidate diagnostic technology for amplifying and measuring the signal of a small quantity of nanometer‐sized markers. The magnitude of the enhanced Raman signal greatly varies with the type of metal, roughness, curvature, and shape of the nanostructure. Research is being conducted on which of these structured substrates efficiently enhances surface Raman scattering. Recently, the medical application of surface‐enhanced Raman analysis has established the groundwork for diagnosis following a label‐free approach, and optimizing the acquired signal has been shown to be possible by controlling the spacing and porosity of nanorod structures when fabricating a SERS chip.^16^, ^17^ Previous studies have demonstrated that animal models of interstitial cystitis and kidney injury were diagnosed by selectively filtering nanometer‐sized biomarkers using a ZnO nanostructure‐based SERS chip.^18^, ^19^ The nanorod‐based surface‐enhanced Raman chip with nanometer spacing proposed in this study has a structure suitable for measuring nano‐sized biological particles such as proteins, lipids, nucleic acids, exosomes, and metabolic substances. If the nanometer marker is used as a diagnostic target, it can be measured in small quantities because it is evenly distributed in the liquid sample, and high reproducibility can be expected. However, as blood consists of a liquid‐based serum and large amount of various cells such as red blood cells, white blood cells, and platelets, selective nanometer‐scale biomarker detection using techniques such as centrifugation entails significant technical time and cost. Because the SERS chip used in this study exhibits nano‐porosity, the chip itself may diffusely separate nanometer‐scale biomarkers from the blood drop without additional separation steps. In addition, as the Raman signal is selectively enhanced by surface plasmons, the excitation is localized to only the separated and trapped region owing to the characteristics of the nanostructure, and processes such as enrichment become unnecessary.^20^

In addition to high‐sensitivity Raman signal acquisition nanotechnology, a technology capable of analyzing these spectral data and securing the basis for diagnosis is required because Raman signals in biological materials exhibit a variety of spectral peaks of various origins. Efforts have been made to secure the basis for diagnosis from data sets consisting of multiple overlapping peaks through dimensionality reduction using techniques such as principal component analysis (PCA). In particular, the Raman signal from a biomaterial has a greater data deviation than that of a crystalline sample, and because each peak of the spectrum changes dynamically, data analysis using automated pattern recognition technology is required. The application of artificial intelligence (AI)‐based automation algorithms to SERS diagnosis technology has attracted considerable attention, as it enables the improvement and optimization of diagnosis accuracy.^21^, ^22^

One method of constructing a mouse model for the implementation of atherosclerosis is by administering a high‐fat diet. However, in wild‐type mice, the generation of plaques seldom proceeds past the early stages of lesion development.^23^, ^24^ In addition, wild‐type mice are unsuitable for generating a model for atherosclerosis alone because other high‐cholesterol‐induced vascular diseases may be involved.^25^ Apolipoprotein E (ApoE) knockout (KO) mice are host to a defective gene involved in cholesterol metabolism that accelerates atherosclerotic plaque formation under a high‐fat diet. In addition, the acute development of atherosclerotic plaques can be triggered by partial carotid ligation surgery, which induces the pro‐atherogenic disturbed blood flow characterized by low and oscillating shear stress.^26^, ^27^ The accelerated rate of atherosclerosis progression in ApoE KO mice minimizes other complications that may accompany atherosclerosis, making them ideal for confirming inflammatory biomarkers or Raman signals caused only by atherosclerosis.

**FIGURE 1:**
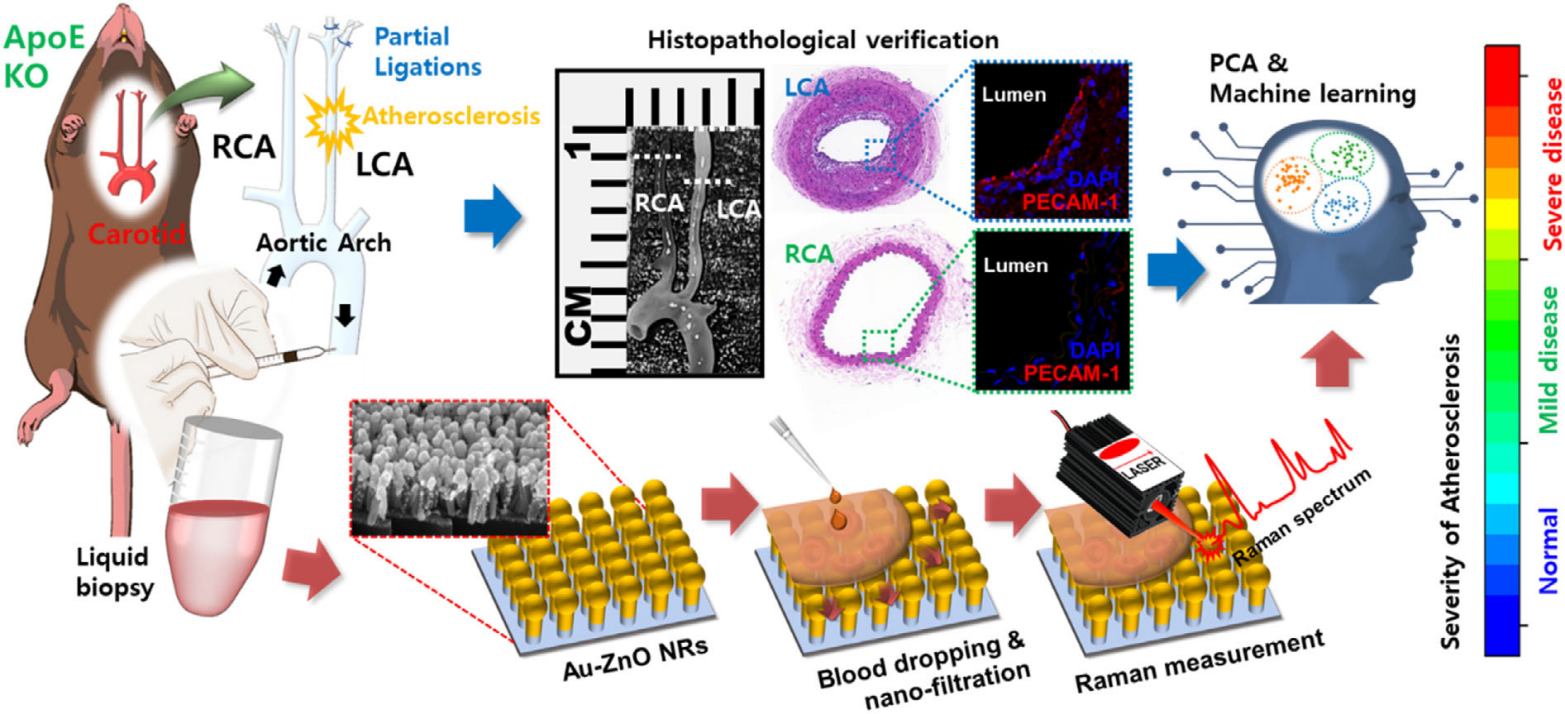
Schematic for nanomarker Raman‐based diagnosis of arteriosclerosis. Atherosclerosis animal model preparation, sample acquisition, histopathology and immunofluorescence staining, pretreatment‐ and label‐free SERS measurement. The red arrows track the process of obtaining a Raman signal based on a nanometer marker for inducing the severity of atherosclerosis, and the blue arrows track the verification process using histopathology. KO, knockout; LCA, left carotid artery; PCA, principal component analysis; RCA, right carotid artery; SERS, surface‐enhanced Raman spectroscopy.

Here, we applied a nanostructured SERS assay to predict early or advanced carotid atherosclerosis development status in individual mice, as shown in Figure 1. A mouse model of disturbed blood flow‐induced atherosclerosis was established by the partial ligation of the left carotid artery (LCA) in an ApoE KO mice that were fed a high‐fat diet. Blood samples were deposited on SERS‐signal‐enhancing gold‐ZnO nanorod chips, and the resultant Raman spectrum was analyzed using PCA, which demonstrated a clear distinction between the disease and control groups. Severe and moderate disease groups were established via concurrent imaging of the LCA by magnetic resonance imaging (MRI) which allowed in vivo monitoring of atherosclerosis progression in the mice, while histological analysis of excised arteries using killed mice further validated the atherosclerosis development. This enabled the development of an early diagnostic criterion based on the severity of atherosclerosis progression, which was based on analyzing statistical information from the Raman signal to determine relevant factors. The criterion's predictive ability was validated by diagnosing an additional group, whose data had not been used to establish the criterion. In addition, a performance review and diagnosis optimization were performed by comparing the performance of PCA to PCA‐PLS‐DA, an additional machine learning algorithm. Altogether, the results indicate that the combination of machine learning and SERS‐based nano biomarker technologies using blood samples merits further application as a means for the early diagnosis of atherosclerosis.

## 2 RESULTS

### 2.1 Selection of atherosclerotic animal groups for diagnostic criteria

**FIGURE 2:**
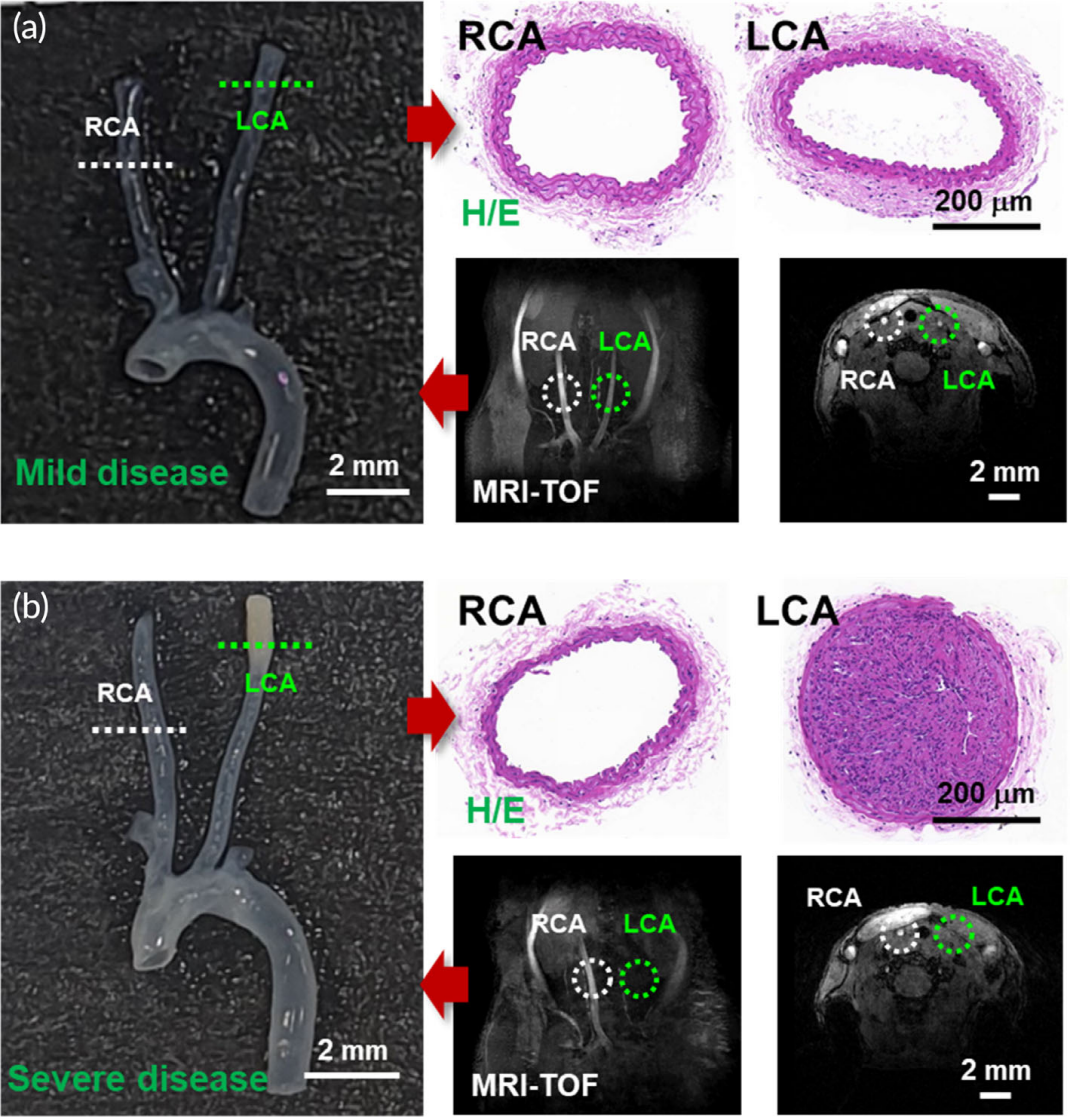
Atherosclerotic blood flow validation and histopathology. Confirmation of blood flow and atherosclerosis using MRI‐TOF and atherosclerotic tissue sections from representative (a) mild and (b) severe disease samples. Tissue sections were taken from the atherosclerotic left carotid artery (LCA) and compared with the healthy right carotid artery (RCA). Histopathological images were stained with H&E. The MRI‐TOF images are shown in the horizontal and vertical cross‐sections. H&E, hematoxylin/eosin; MRI‐TOF, time‐of‐flight magnetic resonance imaging.

To confirm the performance of SERS, atherosclerotic mice were produced by the partial ligation of the LCA, which can generate disturbed blood flow with low endothelial shear stress, as described in previous studies.^26^, ^27^, ^28^ In C57BL/6J ApoE KO mice, three out of four branches of the LCA (left external carotid, internal carotid, and occipital artery) were surgically ligated near the carotid artery, and a high‐fat diet was administered for either 2 or 4 weeks. These mice were measured via MRI to monitor blood flow in the LCA and right carotid artery (RCA). Before extracting carotids from the 2‐ and 4‐week groups, blood was collected from the outlet of the aortic arch and measured using Raman spectroscopy. Figure 2 shows histopathology tissue cross‐section and micro‐MRI blood flow images of the RCA and LCA. In particular, Figure 2b clearly shows that the atherosclerosis is accelerated in the LCA, that the blood vessels are blocked, and that the reduced blood flow of the LCA is visible on the MRI. Based on the presence of LCA and RCA blood flow in the MR image just before carotid extraction, transparency of the extracted carotid, and cross‐sectional histopathology of the LCA, mice were classified into three groups: wild‐type C57BL/6J mice as controls (no LCA ligation), mice without abnormal findings (Figure 2a), and mice with significantly advanced atherosclerosis (Figure 2b). Raman signals were analyzed to identify a diagnostic classifier between these groups.

### 2.2 Microscopic histopathology and immunochemistry of atherosclerotic mouse group

**FIGURE 3:**
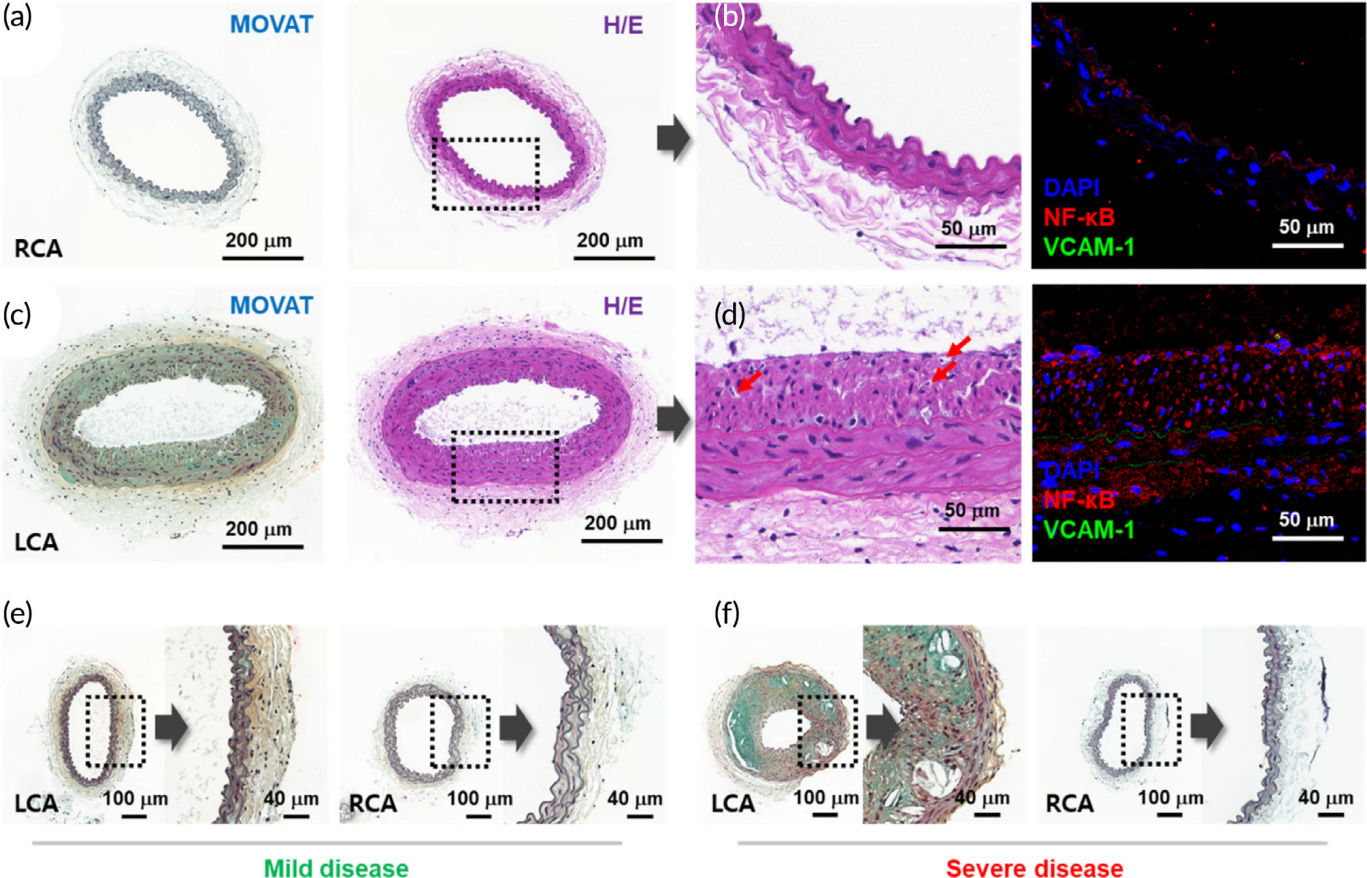
Histopathology and fluorescence of immunohistochemistry. Representative histopathological cross‐sections of blood vessels under accelerated arteriosclerosis (LCA) and in comparative vessels (RCA). (a, c) Movat's pentachrome and H&E staining of (a) RCA and (c) LCA show the atherosclerosis evolution. (b, d) Magnified images for the comparison of the plaque formation and corresponding immunofluorescence confirmation of NF‐κB and VCAM‐1 in (b) RCA and (d) LCA; red arrows: foam cells. (e, f) Movat's pentachrome comparison images of the RCA and LCA in (e) mild and (f) severe disease samples. H&E, hematoxylin/eosin.

Atherosclerosis involves a process of narrowing that occurs as various substances such as fat‐soluble substances and cholesterol in the blood accumulate, starting from the inflammation site on the inner wall of blood vessels. One metric for showing the progress of atherosclerosis is the percentage of the narrowed area in the blood vessel. In particular, the plaque area and progression confirmation in the carotid artery are powerful predictors of high‐risk stroke patients,^29^, ^30^ and have been diagnosed by measuring the plaque burden using intravascular ultrasound.^31^, ^32^ Figure 3a,c is Movat's pentachrome and hematoxylin/eosin (H&E) stained images of the RCA and LCA cross‐sections, respectively. The LCA plaque‐induced turbulence grows internally, resulting in a plaque burden of >78%, as measured according to the plaque area. These plaques in the LCA primarily consist of foam cells (red arrow in Figure 3d) and macrophage‐like cells, and the atherosclerosis is clearly accelerated compared with the RCA. In addition, Figure 3d shows the higher distribution of NF‐kB in plaques formed through ligation‐induced shear stress‐based atherosclerosis induction.^33^ For diagnostic criteria, mice with a plaque burden of 70% or more of the LCA section were selected for the atherosclerosis group, after which Raman spectroscopy was performed. In addition, for where plaque formation was not observed in the RCA but confirmed in the LCA, samples of shear stress or low flow‐induced atherosclerosis through partial ligation were selected as the disease group. Therefore, to obtain diagnostic criteria, the Raman data were divided into “normal” and “mild disease,” which constitute a carotid plaque burden of less than 40%, and “severe disease,” which corresponds to a plaque burden of more than 70%. For mild disease data, almost no plaques were observed to form onto the internal elastic membrane, and the plaque burden was not distinguished by less than 40%, as in the controls. However, when Movat staining was performed, as shown in Figure 3e, a slight increase in fibrin/fibrinoid tissues (reddish) and collagen accumulation (yellowish) were observed in the LCA media area. This parallels the analyses noted by previous studies in atherosclerotic samples,^34^, ^35^ and Figure 3f shows that this increase appears accelerated for severe disease. The samples selected as a mild disease in this study are representative of the early status of atherosclerosis. The mouse groups thus separated were physically induced to produce plaques from blood shear stress through ligation while feeding ApoE KO mice of the same lineage the same high‐fat diet. Chemical additions were controlled so that chemical changes in the blood and changes in biomarkers occurring as plaques were formed could be monitored by Raman.

### 2.3 Utilization of a sensing chip to measure pretreatment‐free nano biomarkers

**FIGURE 4:**
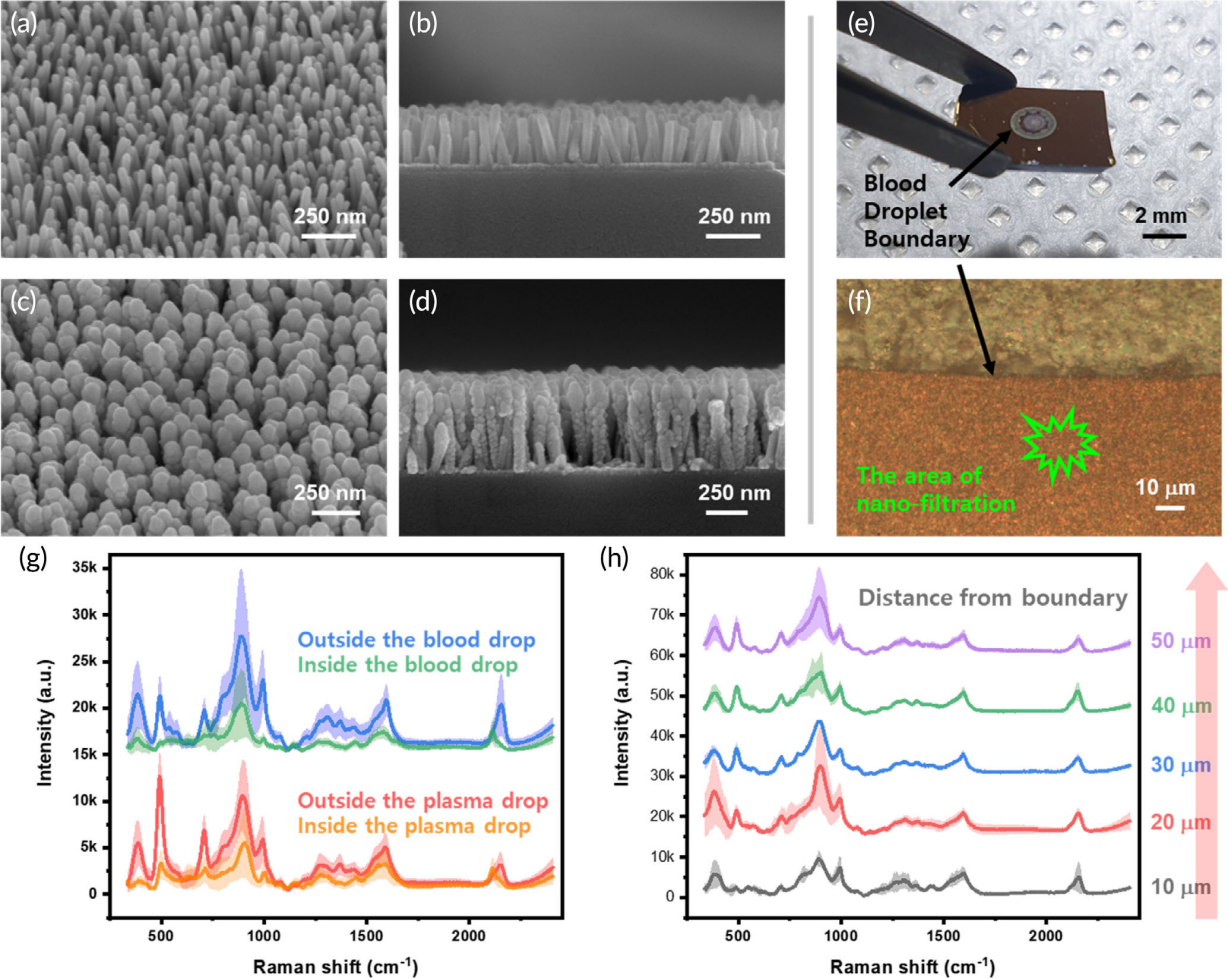
Fabrication, nanofiltration, and test blood measurement of SERS chip. Morphology of SERS chips before and after deposition of pretreatment‐ and label‐free biological samples. (a, b) Cross‐sectional SEM morphology of ZnO nanorods before gold deposition (a) at a 45° angle and (b) perpendicular to the surface. (c, d) Similar conditions to (a) and (b) but following the gold deposition. (e) The same chip, but following the deposition of a blood sample, and (f) with the region of enhanced Raman signal demonstrating the nanofiltration effect of the substrate. (g) Signal comparison of whole blood drop and plasma between the original drop area and filtrated area. (h) Signal difference among measurement distance from the boundary. The average is plotted as a line and the standard deviation as a lighter shade. SEM, scanning electron microscope; SERS, surface‐enhanced Raman spectroscopy.

A ZnO nanorod structure was introduced to the nanoporous structure for the selective filtering of nano biomarkers. By controlling the precursor, temperature, and fabrication time for nanorod synthesis, conditions with an interval within 100 nm were secured, as shown in Figure 4a,b. To enhance the Raman signal through surface plasmon resonance, gold was deposited on the ZnO structure to fabricate the SERS chip shown in Figure 4c,d. The deposited gold maintains gaps of several tens of nanometers in the form of grains, and nano biomarkers in the blood are diffused and located between these gaps. Figure 4e shows a blood drop dried on the SERS chip of this study. The blood drop maintains a spherical shape owing to surface tension, and only nanometer markers are diffused and flowed between the nanogaps. Figure 4f is a microscope image showing the droplet boundary, and the Raman spectrum is obtained by irradiating a laser to the outside point of the boundary (indicated in green).

To evaluate the signal difference between the filtered area and the original droplet area, Raman spectroscopy signals were measured using whole blood and centrifuged blood plasma drop. Figure 4g shows the comparison of the Raman signal inside and outside the droplet boundary, and it is shown that the signal rises dramatically in the outside area (nano biomarker filtrated area). In addition, because there seems to be a rare difference in signal between whole blood and centrifuged plasma, the SERS sensing chip used in this study does not require sample pretreatment such as centrifugation and heparin treatment. Because signal acquisition is possible in one drop, only a very small amount of blood is required compared to the amount required for centrifugation, so there is rare animal kill or damage, even in mouse experiments. Figure 4h shows the difference of the Raman signal by distance at the droplet boundary and shows no variation dependence according to the distance in the corresponding range. The difference between the inside and outside of the droplet boundary was plotted as the average and standard deviation for every 20 points, and the difference according to distance was the result for every 5 points. On the other hand, when the Raman signal was acquired in the whole blood‐filtered area using a 532‐nm laser, the Raman peak pattern disappeared due to strong noise caused by auto‐fluorescence.

### 2.4 Raman spectra and assignments in atherosclerotic blood

**FIGURE 5:**
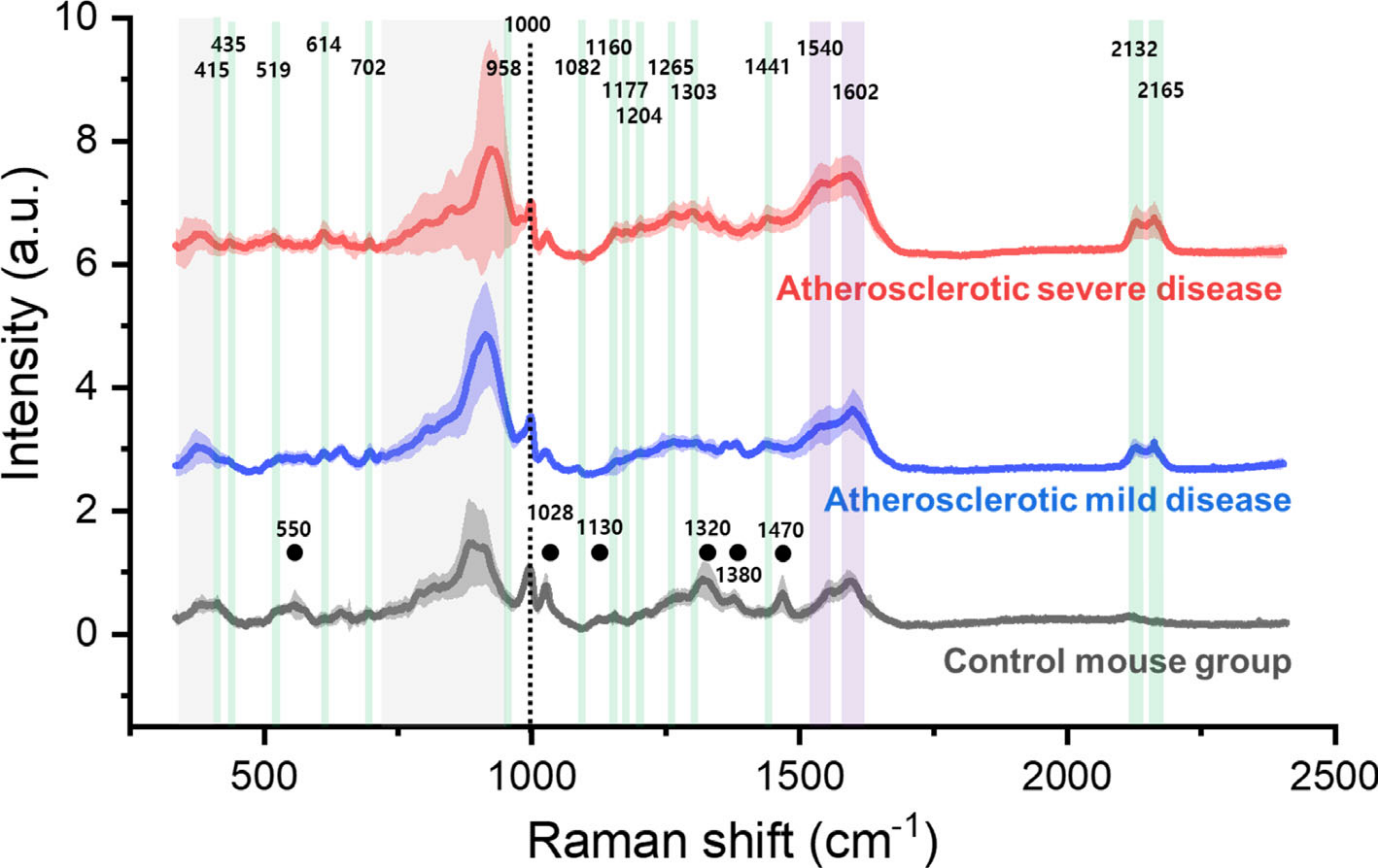
Raman signals by animal group and labeling by major peaks. Raman spectra of blood sampled from control group C57BL/6 wild‐type mice (black), mice with mild atherosclerotic disease (blue), and mice showing severe atherosclerosis (red). Each solid‐colored line shows the average value of the measured spectra, with lighter shades showing the standard deviation. Spectra were normalized based on the value at 1000 cm^−1^ (black dotted line). Black dots identify peaks suppressed as atherosclerosis progresses. Green bands indicate peaks that emerge as atherosclerosis progresses.

Figure 5 shows the average Raman spectra of the selected normal mouse and of the atherosclerotic mild and severe disease mouse groups derived from ApoE KO mice. Solid‐colored lines indicate the average value of the measured spectra, and the corresponding lightly shaded regions indicate their standard deviations. The peak corresponding to 1000 cm^−1^ was assigned to phenylalanine, which is an amino acid present at high concentrations in the body; normalization was performed based on this value. The black dots indicate the main peaks of the spectrum graph that disappeared in atherosclerosis mice when compared with normal mice. Remarkable peaks in ApoE KO‐based atherosclerosis are highlighted with translucent bars, and Table 1 summarizes the assignment of the Raman peaks. Peaks corresponding to the green translucent bars are determined from cholesterol and cholesterol esters, and the standard deviation is small, indicating reliable reproducibility. Moreover, the data variation is large in the ~400 and 730–970 cm^−1^ region (gray bar), which has a wide distribution in terms of both inter‐ and intra‐animal, making a meaningful analysis of peak assignments difficult. Details of the contribution and independence of the statistical analysis for the diagnostic criteria for atherosclerosis in this spectral region are described separately.

**TABLE 1:** Raman signal assignments of atherosclerosis of ApoE KO mice.

Peak	Assignment	Sign in PC1 eigenvector (+/−)	Refs.
415	Phosphatidylinositol	−	36
435	Cholesterol ester	+	36, 37
519	Phosphatidylinositol		36, 38
548	Cholesterol	−	36, 38
614	Cholesterol ester	+	36, 38
702	Cholesterol ester		37, 39
872	Lipid	−	38
958, 1177	Myristic acid in fatty acid	+	37
1000	Phenylalanine		38
1028	Phenylalanine		40, 41
1082	Carbohydrate residues of collagen	+	38
1131	Fatty acid		36
1160, 1204	Tyrosine	+	38, 42
1265, 1303	Cholesterol ester	+	36, 37
1320	Amide III (protein)	−	38, 39
1380	CH_2_ and CH_3_ band (lipids and proteins)	−	43
1441	Lipids (accumulated in the necrotic nucleus of the atheromatous plaque)	+	36, 37, 44
1470	CH_3_ asymmetrical bending of lipids		45
1540	Amide II	+	38, 46
1602	Phenylalanine	+	38, 47
2132, 2165	CN stretching‐related vibration	+	48, 49, 50

Abbreviations: ApoE, apolipoprotein E; KO, knockout; PC, principal component.

### 2.5 Diagnostic criteria for atherosclerosis based on PCA of Raman signals

**FIGURE 6:**
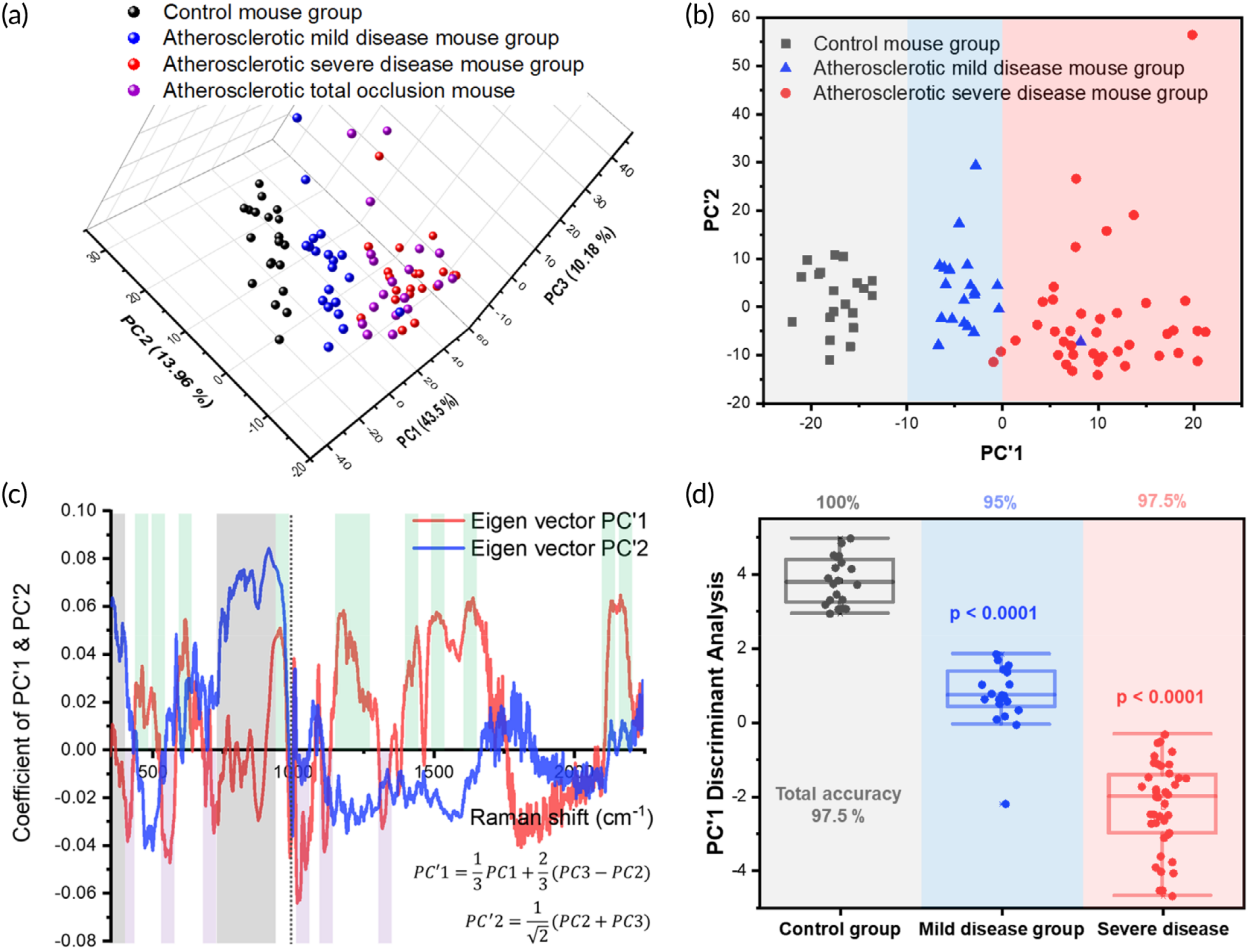
Statistical analysis using principal component analysis (PCA). The principal components of Raman spectra were calculated to form a set of eigenvectors with diagnostic validity for atherosclerosis. The data projected into (a) the three‐dimensional space of the first three principal components and into (b) the two‐dimensional space of the reconstructed principal components. Data are colored to indicate the severity of atherosclerosis: control samples: black, mildly atherosclerotic samples: blue, and severely atherosclerotic samples: red. Samples with clogged blood vessels are shown in purple. Colored bands indicate linear boundaries between classifications based on PC1. (c) Reconstructed graph of PC eigenvectors and derivation formulae. Regions of positive contribution to PC1 are shaded with green bands, whereas negative contributions are indicated by purple bands. (d) Accuracy and significance for each group based on PC1 values. The horizontal line inside the box for each group indicates the mean, and the horizontal line outside the box indicates the deviation.

PCA determines the correlated variation between variable sets. By treating each point in the spectrum as a variable, PCA extracts the correlated variability in the spectrum caused by the several different vibrational states of individual nanoparticles and vibrations of different nanoparticles that frequently appear together. In addition to improving the signal, PCA allows the dimensionality of the data to be reduced to the dimensions with highest variance, which are the principal components (PCs). PCA was performed for normal, mild atherosclerotic, and severely atherosclerotic Raman signals. The inter‐ and intra‐animal effectiveness was verified by analyzing 20 points for each group and 5 points per animal for four animals. In addition, for the severe disease group, the results shown in Figure 6a were obtained by labeling the plaque burden that increased by 70% or more, as shown in Figure 3c, as well as the blocked blood vessels shown in Figure 2b. The variability captured by the principal components was 43.5% for PC1, 13.96% for PC2, and 10.18% for PC3. Projecting the experimental data along the first principal components highlights this variance. Among the viewing planes for the projection of the data into the three‐dimensional space of PC1, PC2, and PC3, a viewing plane showed the arrangement and separation of the data in the order of normal, mild, and severe atherosclerosis, as shown in Figure 6a. The projected plane is expressed as the two‐dimensional plane of PC1 and PC2, as shown in Figure 6b, and the evolution of atherosclerosis in the PC1 direction is coherently well‐shown. Figure 6c provides the definitions of PC1 and PC2.

Diagnostic criteria were prepared according to the projection in the PC1 direction, with a score of −10 or less denoting the region with mostly control samples, between −10 and 0, the region of mild disease samples, and 0 or more indicating severe disease. As shown in Figure 6d, the total accuracy according to these criteria was calculated to be 97.5%. The significance for each of the mild and severe disease groups was expressed as a *p* value, and each *p* < 0.0001 was confirmed. In addition, the diagnostic validity according to PC1 was reviewed using the machine learning technique used in the application of diagnostic statistics for the recognition of Raman signals.

### 2.6 Machine learning‐based validation of PCA diagnostic criteria

**FIGURE 7:**
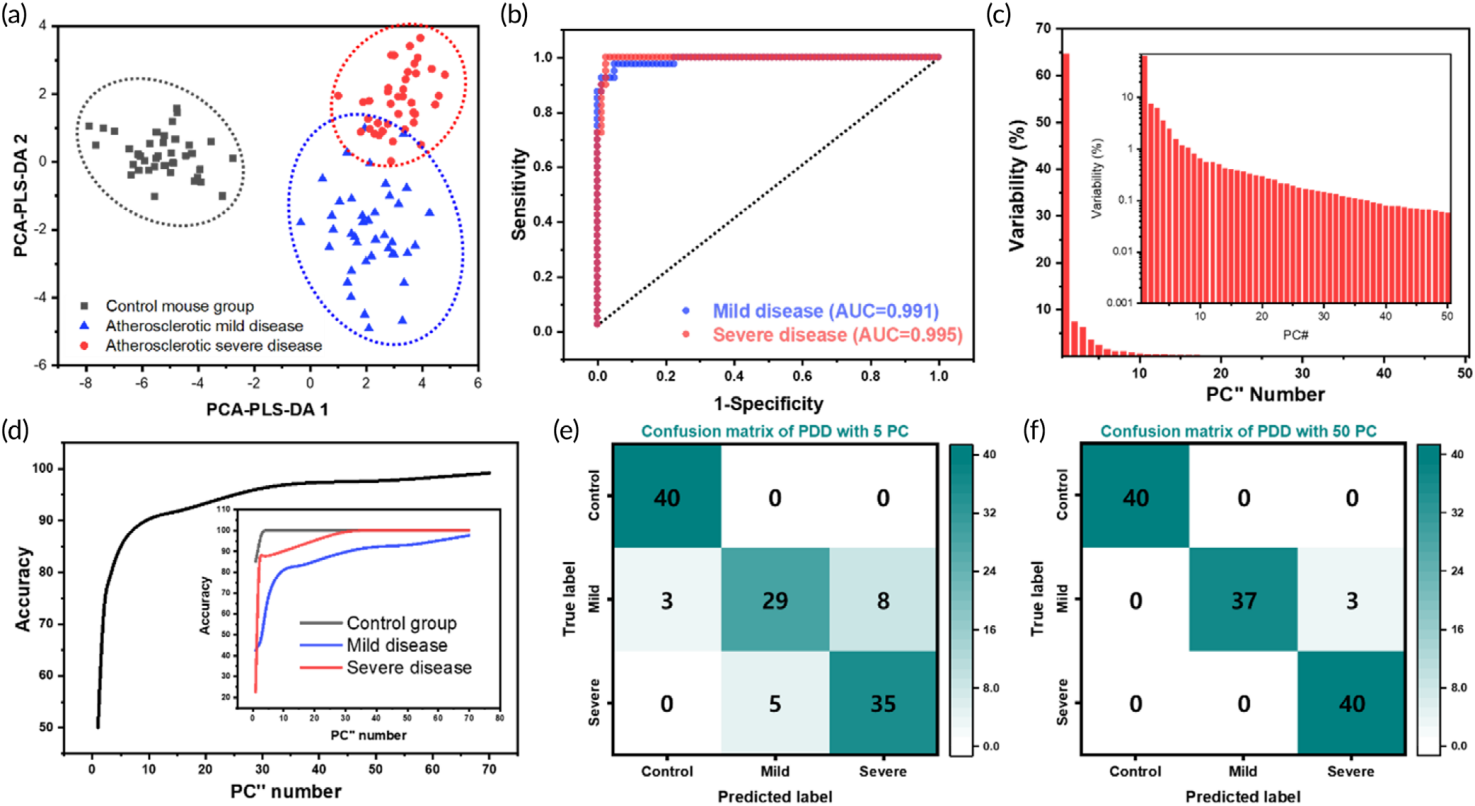
Analysis using machine learning algorithm of PCA‐PLS‐DA. (a) Data distribution when the PLS‐DA machine learning algorithm is used with values up to PC50 as variables. (b) Receiver operating characteristic (ROC) curve and area under the curve (AUC) values for mild and severe disease groups, respectively. (c) Distribution of variability from PC1 to PC50, with the corresponding log scale, inset. (d) Total accuracy curve of PCA‐PLS‐DA according to the number of selected PCs. Inset shows accuracy for each group. (e, f) Confusion matrix for the PCA‐PLS‐DA classification with 5 and 50 PCs as variables, respectively. DA, discriminant(s) analysis; PCA, principal component analysis; PLS, partial least squares regression.

PCA‐partial least squares regression (PLS)‐discriminant analysis (DA) modeling is a machine learning algorithm useful for clustering spectral components in Raman spectroscopy.^21^ Using PCA‐PLS‐DA modeling, the control, mild, and severe groups were confirmed to be divided as shown in Figure 7a. In particular, the PLS‐DA approach generally performed better in finding the input variables that have the closest relationship to the output variables than in PCA, which simplifies the data set in Raman.^51^, ^52^ To validate the performance, the PLS application was conducted using 50 principal components calculated from 40 samples from each of the control, mild, and severe atherosclerotic disease mice. Variable reduction was performed through PCA, and the accuracy was evaluated by applying PLS‐DA. The data distribution through PCA‐PLS‐DA is shown in Figure 7a, and Figure 7b shows the receiver operating characteristic (ROC) curve. The accuracy of diagnosis measured from the area under the curve (AUC) was 99.1% and 99.5% for mild and severe disease, respectively. Figure 7c shows the distribution according to the PC variability. Depending on the number of PCs selected as variables, the accuracy of PCA‐PLS‐DA shows the curve as in Figure 7d, and the difference in accuracy by animal group is shown in the inset. The confusion matrix when 5 and 50 PCs were selected and analyzed is shown in Figure 7e,f, respectively. Although it improved by about 3% compared with the accuracy derived by PC1, it already shows high accuracy in PCA‐based measurements, which will increase the usefulness of machine learning analysis for additional variables and applications to clinical samples. As shown in Figure 7a, PCA‐PLS‐DA1 and 2 are insufficient compared with PC1 in distinguishing the control, mild, and severe data with a single vector. In this study, the Raman signal of the arteriosclerosis nano biomarker sufficiently differs for each group to show a good accuracy even in PCA‐based data separation. In addition, PCA is the most commonly used statistical method for determining the principal components that best explain differences in spectra.

### 2.7 Principal component eigenvectors provide high contribution to diagnostic criterion

As shown in Figure 6b, atherosclerosis proceeds in the positive direction of the PC1 vector; thus, the coefficients of this eigenvector show the parts of the Raman spectra that have significant correlations with diagnostic meaning. In Figure 6c, the eigenvectors for PC1 and PC2 are plotted as coefficient values according to the Raman energy shift. In addition, the direction parallel to PC2 and positive is observed to be independent of the sample group boundary in Figure 6b. In Figure 6c, the spectral region that contributes the main value to the PC2 positive direction is indicated by translucent gray bars. This region extends up to 425 cm^−1^ and assumes a high value in the range of 710–980 cm^−1^. While this range corresponds to a wide region of high spectral intensity in Figure 5, this area is a dead space with minimal diagnostic meaning, as shown by its low (and mostly orthogonal) value in the PC1 eigenvector.

In Figure 6b, the PC1 scale was normalized such that the normal group is divided at a value of −10, and the mildly and severely atherosclerotic disease groups are divided at 0, in the PC1 direction. Therefore, the stronger the PC1 eigenvector and spectrum values in the positive direction, the closer the sample is to (severe) atherosclerosis. Table 1 presents the positive and negative contributions of the eigenvector according to the spectral peak assignments. Most positive contributions were from cholesterol ester (430–515 and 577–680 cm^−1^); cholesterol, cholesterol ester, and tyrosine (1140–1305 cm^−1^); lipids associated with atheromatous plaque (1390–1460 cm^−1^); amide II (1480–1580 cm^−1^); phenylalanine (1600–1710 cm^−1^); and CN stretching vibration (2115–2215 cm^−1^), indicated by a green transparent bar in Figure 6c. Moreover, the main factors contributing to the weight in the negative direction include peaks corresponding to phosphatidylinositol (380–430 cm^−1^), cholesterol (520–580 cm^−1^), phenylalanine (1010–1070 cm^−1^), and amide III (1305–1350 cm^−1^). The negative value of the eigenvector means the weight of the corresponding substance (peak intensity) for atherosclerosis diagnosis and is far from the increase or decrease of the absolute value of the substance. In addition, because the analysis was based on data normalized to 1000 cm^−1^, the difference in relative values becomes the criterion for diagnosis.

### 2.8 Validation of classification criteria using data from additional animal groups

**FIGURE 8:**
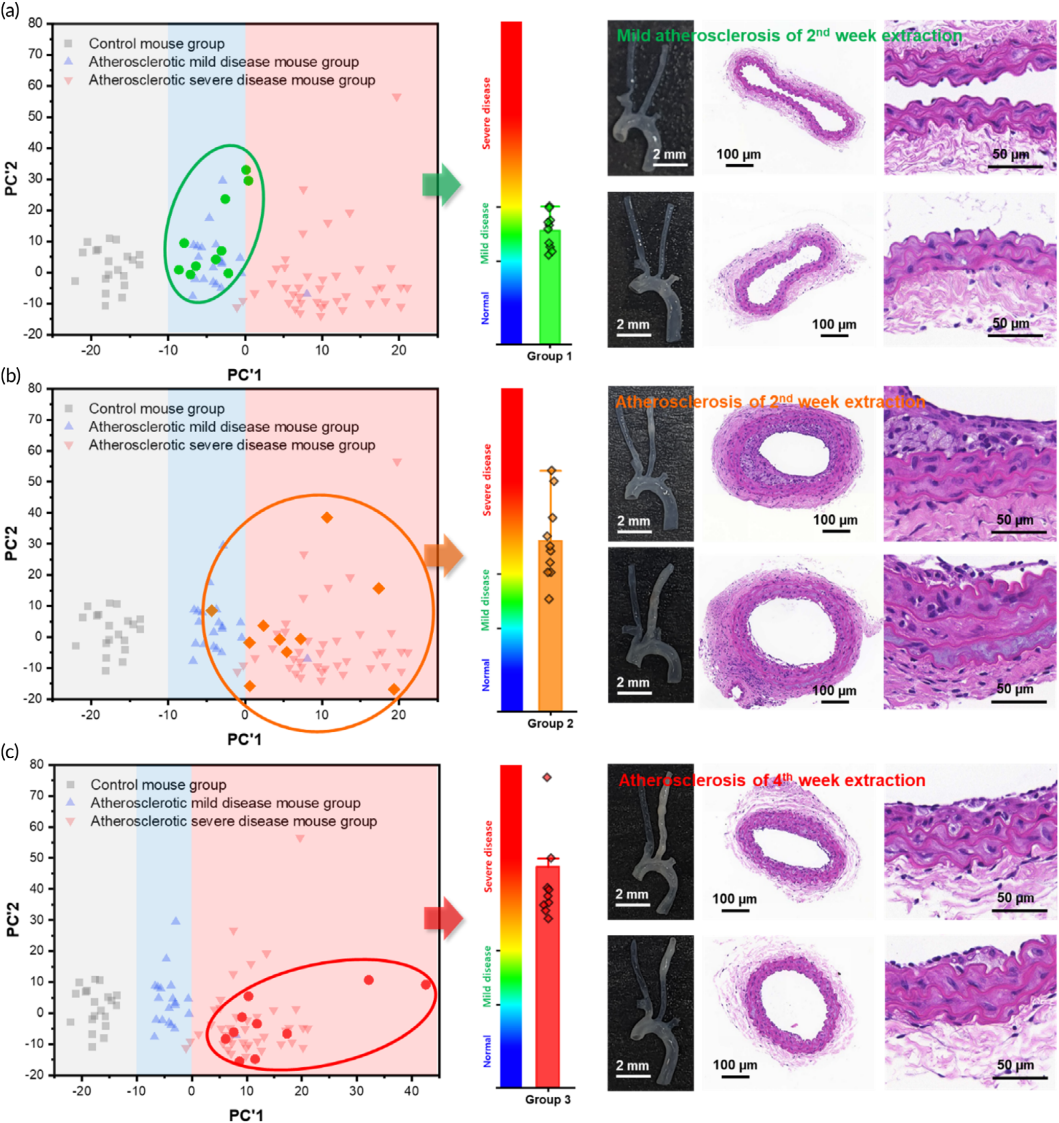
Validation of diagnostic criteria using additional Raman data. Diagnostic accuracy was validated by performing a diagnosis on additional data using the diagnostic criteria from Figure 6b. The original training data points used to establish the diagnostic criteria are blurred in the background. For each group, the bar chart represents the severity of atherosclerotic samples, as defined according to PC1. (a) Distribution of Raman data and representative histopathological results from ApoE mice with confirmed blood flow 2 weeks after carotid artery ligation (mild atherosclerosis group). (b) Data from mice with clear atherosclerosis progression after the second week following ligation. (c) Data from atherosclerotic mice at the fourth week following ligation. PC, principal component.

Machine learning algorithms may be used to create diagnostic models. However, owing to the risk of overtraining, these models cannot be considered meaningful until tested on an independent data set. The validity of the atherosclerosis diagnosis secured based on the eigenvectors and Raman assignments in Figure 6b was assessed using data from additional animal groups. Figure 8 shows the projection of this new data into the space of the PC1 and PC2 eigenvectors by applying the same normalization conditions to insert additional Raman data into the PC space of the diagnostic criteria. The data added are shown with bright colors, and previous data points are shown in pastel colors in the background for context. The added Raman data sample group was obtained from the blood of ApoE KO mice with partially ligated LCAs in the second and fourth week after ligation. Based on histopathological results, the data from the second‐week sample group with mild atherosclerosis progression are distributed, as shown in Figure 8a. The PCA results were visualized by reconstructing a bar chart that compares each validation group with the previously defined diagnostic criteria. The data from the second‐ and fourth‐week animal groups indicate a narrowing of blood vessels and plaques on histopathology distributed in the atherosclerotic region of the diagnostic criteria, as shown in Figure 8b,c.

## 3 DISCUSSION

Atherosclerosis is initiated by inflammation in the endothelial layer of the arterial wall, which allows the endothelium to become more permeable. After the barrier of the endothelium is compromised, bloodborne LDLs accumulate in the subendothelium, where they are oxidized by macrophages and smooth muscle cells, resulting in the conversion of macrophages to foam cells. The release of various cytokines by the dysfunctional endothelium leads to the recruitment of circulating monocytes and transmigration by binding to endothelial cell adhesion molecules, which is followed by foam cell accumulation, smooth muscle cell migration and proliferation, and finally, the formation of fibroatheroma containing necrotic core in the advanced atherosclerosis stage.^53^, ^54^

The sites likely to support atherosclerotic progression are the outer wall of vessel bifurcations, inner side of curved vessels, and walls of vessels downstream of the stenotic segments, which are characterized by disturbed blood flow with low and oscillatory shear stress.^55^, ^56^, ^57^ Wall shear stress, which is the frictional force between the blood and endothelium, is a major determinant of endothelial function and regulates mechanosensitive gene expression. Both mechanosensing and mechanotransduction are fundamental physiological mechanisms allowing the endothelial cells to react to hemodynamic forces.^58^, ^59^

Partial carotid ligation is a novel method that enables the collection of endothelial‐enriched RNAs and evaluation of the molecular mechanisms underlying flow‐dependent regulation in vascular biology. A previous study associated the disturbed flow site generated by partial carotid ligation with endothelial dysfunction, the regulation of pro‐ and anti‐atherogenic genes, and rapid atherosclerosis progression.^28^ In this study, atherosclerosis was selectively induced in the LCA via partial ligation of carotid arteries in high‐fat‐diet ApoE^−/−^ mice. Through histopathology with Movat pentachrome, H&E, and immunofluorescence (IF) staining, foam cell formation in the plaque and an increase of related cytokines and collagen were confirmed. Under the controlled conditions of the animal model, biomarkers or molecular biological information directly contributing to atherosclerosis were expected in the blood.

To monitor existing carotid atherosclerosis from markers circulating in the blood, diagnoses have been performed using several biomarkers, including inflammation‐based markers, cellular molecules, lipids, leukocyte counts, and cytokines.^1^, ^2^, ^7^ Such diagnoses require a lot of time, and selectively measuring a target marker through labeling involves many steps, including separation, culture, labeling, and detection. In contrast, in this atherosclerosis diagnosis technology, sample separation occurs as samples diffuse through nanostructures and signals are selectively enhanced through surface plasmon effects. On the nanostructured ZnO surface, these naturally occur within a few minutes from depositing a drop of blood. While nanometer markers are difficult to detect in blood because of their small size, low density, and sporadic distribution, they are distributed in the serum to be consistently detectable.

Surface‐enhanced Raman technology is possible because it amplifies the signal intensity by a factor of 10^5^ or more where the nanometer‐scale markers are trapped. However, selectively measuring only nanometer‐sized markers has limitations; for example, it does not provide information on changes in leukocyte (approximately 10 μm in diameter) counts or the composition of macrophages. In addition, the present study is limited in its clinical applicability; while Raman spectra are predictive for arteriosclerosis among the study population, some arteriosclerosis biomarkers are known to be markers for systemic inflammation.^2^ The present diagnostic technique has not been demonstrated to distinguish between arteriosclerosis and other inflammation; thus, the model likely requires tuning to achieve diagnostic specificity in a clinical environment.

Nonetheless, this study shows that distinguishing between normal and atherosclerotic blood is possible along with determining the severity of atherosclerosis progression from a single drop of blood in animal models, with only nanostructure‐based SERS. In these models, diagnosis was correlated with the assignment of vibrational peaks to known nanometer‐scale markers, including cholesterol esters, lipids, and carbohydrate residues of collagen in the obtained enhanced Raman signal. This nanomarker Raman sensing shows consistent differences in the signals even in mild atherosclerosis disease, in which plaque progression is rare. The accuracy of the diagnosis was confirmed from the increase in fibrin/fibrinoid and collagen in vascular Movat staining, which are associated with arteriosclerosis. This nanometer marker‐based Raman analysis platform was derived using PCA statistical analysis, and the performance of this analysis capability was verified using a machine learning algorithm. Considering the basis for securing the diagnosis, the detection‐based diagnosis of nanometer markers from blood obtained from clinical patients is also expected to be effective. In addition, the risk of arteriosclerosis can be visualized in the form of a bar chart, as shown in Figure 8, for a medical examination in the clinical field. This shows that nanomarker‐based Raman detection is an attractive approach for the development of diagnostic technology that can diagnose the early stage of arteriosclerosis from a drop of blood.

To secure the clinical validity of the results of this study, which diagnoses arteriosclerosis with a single blood drop, it seems important to solve the problems in two aspects. First, unlike animal experiments, variation in patient‐derived blood will be large, and selection of the patient's control group is important. Among people undergoing medical check‐ups in a health service and promotion center, asymptomatic subjects who have low cardiovascular risk and show normal findings on carotid ultrasound (or MR angiography) and coronary CT angiography will be enrolled in the control group. Second, advanced atherosclerosis involving cerebral, coronary, and peripheral arteries may lead to stroke, ischemic heart disease, and peripheral vascular disease, respectively. Further study is needed to verify if the SERS signals generated by atherosclerotic process are organ‐specific. If ischemia‐producing severe stenosis is detected earlier by the Raman‐based method, both medical and interventional treatment may improve clinical outcomes. In a previous study, ischemic renal dysfunction was detected with the SERS sensing chip,^60^ and this Raman signal and the Raman signal in atherosclerosis were separated. This shows that it is possible to diagnose organ‐specific atherosclerosis, and there are plans to expand the scope through various animal and clinical studies in the future. Accordingly, it seems that machine learning through data accumulation will be required, and the machine learning algorithm used in this study will accelerate the successful achievement of the research achievement. If diagnostic criteria are secured through data accumulation, in patients with mild to moderate disease, appropriate lipid‐lowering therapy and lifestyle modification would prevent atherosclerotic acceleration and reduce the rate of major adverse cardiovascular events.

## 4 CONCLUSION

In summary, through the selective Raman spectroscopic detection of nanometer markers, technology has been developed for the diagnosis of early stage acute atherosclerosis from a single blood drop. Animal blood samples for an atherosclerosis diagnostic baseline (training set) were prepared using carotid artery ligation of ApoE^−/−^ mice which were administered a high‐fat diet. Classifications for establishing the diagnostic baseline were based on the severity of atherosclerosis as observed through micro‐MRI and histopathology (Movat pentachrome, H&E, and IF staining). Blood from each animal group was collected, 5 μL of which was dropped on a nanoporous sensing chip, and a 785‐nm laser was irradiated on the area where the sample had diffused through the porous structure to obtain a spectrum of the surface‐enhanced Raman signal scattered from nanoscale biomarkers. The Raman signal was correlated with atherosclerotic severity; the signal was observed to increase in the spectral regions representative of cholesterol ester, lipids, and carbohydrate residues of collagen as atherosclerosis progressed, and PCA was performed using the Raman spectra and showed groupings of samples within the control, mild, and severe arteriosclerosis groups. Control and atherosclerosis data were distributed and grouped in the direction of the first principal component to establish a standard for atherosclerosis diagnosis. From these diagnostic criteria, the diagnosis of additional animal groups was verified to ensure validity. Furthermore, the accuracy of the diagnostic criteria for which this signal basis was secured was 94.5%, which approaches the 97.5% accuracy of data classification obtained using the PCA‐PLS‐DA machine learning algorithm. While not establishing specific biomarkers, this indicates the existence of early‐stage diagnostic biomarkers representative of atherosclerosis in the blood of mice with acute flow‐induced arteriosclerosis; the existence of detectable multifactorial biomarker diagnostics can hopefully be generalized to human patients with arteriosclerosis due to a wide variety of etiologies.

## 5 METHODS

### 5.1 ApoE KO‐based atherosclerosis animal model

All animal experiments were conducted under the guidance and regulations of the Asan Medical Center Institutional Animal Care and Use Committee (IACUC). Two mice groups were established: a control group of male C57BL/6 wild‐type mice and treatment group of male C57BL/6 ApoE^−/−^ mice, both purchased from Jackson Laboratory. In the high‐fat diet of C57BL/6 mice, there were differences in HDL levels according to male or female.^23^ In this study, it was fixed as male to minimize variation in blood biomarkers by sex. The generation and evaluation of mice carrying the inactivated mutant apolipoprotein E gene were confirmed.^61^ At 8 weeks of age, the carotid artery was ligated in the treatment group to produce a disturbed blood flow (d‐flow), which is known to lead to consistent and representative generation of arteriosclerosis in these mice.^26^, ^27^, ^28^ For the arterial ligation, a mouse was placed under respiratory anesthesia and injected with an analgesic. Respiratory anesthesia was maintained during surgery using 2.0%–2.5% isoflurane in a 1:2 mixture of O_2_:N_2_O delivered through a mask. Surgery consisted of depilating the chest, making an incision in the center of the chest, and identifying the carotid artery. All three major branches of the LCA (external carotid artery [ECA], internal carotid artery [ICA], and occipital artery [OA]) were individually ligated with 6‐0 black silk suture, and the incision was closed. After 3 days of observation, the analgesic was injected. A high‐fat diet (VHFD 60 kcal% fat #D12492, Research Diets) was used to generate atherosclerosis in the treatment group, and mice were divided into smaller groups for 2 and 4 weeks of dietary intervention (*n* = 20 each). The high‐fat diet began immediately after surgery. MRI was performed once on the day after surgery and again the week before killing. Under anesthesia with Zoletil (Zoletil50), blood was collected from the inferior vena cava, and the mice were then euthanized. The vascular tissue in which atherosclerosis was induced was obtained and refrigerated in 4% formaldehyde (Biosesang) at 4°C for follow‐up staining and histology.

### 5.2 Blood flow through TOF‐MRI

MRI was conducted in time‐of‐flight (TOF) mode using a 9.4 T/160 mm animal MR system (Agilent Technologies). Excitation was performed using a 72‐mm birdcage volume coil, and a two‐channel phased array surface coil was used for signal reception. Seven repetitions were averaged, and the fat sat short pulse feature was used to amplify fat detection. Before imaging, mice were anesthetized with 2.0%–2.5% isoflurane in a 1:2 mixture of O_2_:N_2_O delivered through a mask. Respiration was monitored, and mice were maintained at an environmental temperature of 37.5 ± 0.5°C using an air heater system.

### 5.3 Histochemical evaluation and IF staining for atherosclerosis sample group

The atherosclerotic vascular tissue was subjected to histochemical evaluation, and each cross‐section slide of carotid was prepared into 4‐μm thick slices. H&E and Movat pentachrome stain kits (BioGnost Ltd.) were used to stain vascular tissue slides per the manufacturer's protocols for histochemistry of atherosclerotic progression. Movat staining indicated nuclei and elastic fiber in black, collagen and reticulin fibers in yellow, mucins in blue to green, and fibrins and muscle fibers in red. All histochemical slides were digitized with the Panoramic 250 Flash I digital slide scanner (3DHISTECH) at ×20 magnification.

To measure the plaque area, the lumen and external elastic membrane (EEM) area were measured. The plaque area was calculated as [EEM area − lumen area]; the plaque burden was calculated as the plaque area divided by EEM × 100 (%).

Sample slides for fluorescent immunohistochemistry were deparaffinized in Xylene (Junsei Chemical) and washed. The primary antibody for NF‐κB (Anti‐NF‐κB p65, Abcam) and VCAM‐1 (VCAM‐1 Monoclonal Antibody, Invitrogen) were applied, a green secondary antibody (Alexa Fluor 488, Invitrogen) was then labeled for VCAM‐1, and a red secondary antibody (Alexa Fluor 555, Invitrogen) was labeled for NF‐ κB. Samples were washed with distilled water and embedded in a mounting medium after DAPI treatment. Fluorescent images were acquired and analyzed using a confocal laser scanning microscope system (LSM780; Carl Zeiss) with ×20 objective lens.

### 5.4 Surface‐enhanced Raman sensing chip fabrication

A 30‐nm ZnO seed layer was deposited on a 6‐in. Si wafer by radio frequency (RF) sputtering at a power of 100 W. The ZnO seed on the Si was blown with N_2_ gas and cut to a size of 9 × 9 cm^2^. Subsequently, ZnO nanorods were grown by hydrothermal synthesis processes. ZnO nanorods were grown from a solution of 25 mM of zinc nitrate hexahydrate (Zn(NO_3_)_2_·6H_2_O, ≥99.998%, Alfa Aesar) and 25 mM of hexamethylenetetramine (HMTA ≥99.5%, Sigma‐Aldrich) in 50 mL of deionized (DI) water heated at 90°C for 60 min. After growing the ZnO nanorods, substrates were washed with DI water and dried with nitrogen gas. Finally, an amount of gold equivalent to a 200‐nm thin film was deposited on the grown ZnO nanorod substrate using an E‐beam evaporator (ULTEC). The morphological and structural properties of the Au‐coated SERS chips were analyzed using a field‐emission scanning electron microscope (FE‐SEM; S‐4700, HITACHI) at a beam voltage of 10 kV.

### 5.5 Statistical analysis and machine learning validation for Raman spectral data

Blood sampled from the inferior vena cava of a random sampling of mice in each group (*n* = 5) was dropped in 5 μL quantities on SERS chips and left until spreading was complete. A Raman spectrometer (NOST) equipped with a ×40 objective lens (LUCPLFLN40X, Olympus, NA = 0.6, WD = 2.7–4.0) and 785‐nm laser source passed through a 1% neutral density filter was used to illuminate and measure Raman spectra from the samples. Raman spectra were measured five times for 4 s each from 400 to 2400 cm^−1^ via a 600 groove grating. The spectral data pitch was 2 cm^−1^ for 1000 data points. Sample measurements were accumulated for 20 s, and five‐polynomial fitting was then applied to remove background noise. To evaluate chip performance through whole blood and centrifugal plasma, 1 mL of mouse blood was placed in PST Tubes with Lithium Heparin (Becton and Dickinson), one drop was supplied to the SERS chip, and one drop of plasma was also dropped onto the SERS chip after centrifugation. Blood plasma separation was performed in a centrifugation system (hanil M15R) at 2500×*g* for 10 min at 4°C.

PCA was employed to identify differences in the Raman spectra of the control and atherosclerosis groups. The entire spectral range was used as a variable, and the analysis was conducted using XLSTAT 2019 (Addinsoft). To validate the PCA diagnostic criteria derived from the Raman spectra, PCA‐PLS‐DA modeling was conducted using XLSTAT 2019. The PCA‐PLS‐DA classifier evaluated the segregation of disease severity with a PLS‐DA classification model using 50 PC‐based variables that accounted for >98.15% of the data set variance. Weights of the first 50 PCA‐based eigenvectors were input into the PLS‐DA classifier as a 50 × 120 matrix, similar to previous work.^61^ This analysis evaluated the performance of the classifier using 120 spectral samples, consisting of 40 from normal mice, 40 from mildly atherosclerotic mice, and 40 from severely atherosclerotic mice as training data. These data were obtained from five, seven, and six mice in the normal, mild, and severe atherosclerotic disease conditions, respectively. The PLS‐DA algorithm outputs an additional set of classifying vectors based on the PCA eigenvectors. From these calculations, the data distribution in the PCA‐PLS‐DA plane and confusion matrix were plotted.

## AUTHOR CONTRIBUTIONS

**Sanghwa Lee:** Data curation (equal); formal analysis (equal); methodology (equal); software (equal); validation (equal); visualization (equal); writing – original draft (equal). **Miyeon Jue:** Data curation (equal); formal analysis (equal); investigation (equal); methodology (equal); software (equal); validation (equal). **Minju Cho:** Data curation (equal); investigation (equal). **Kwanhee Lee:** Data curation (equal); investigation (equal). **Bjorn Paulson:** Formal analysis (equal); investigation (equal); validation (equal); writing – original draft (equal). **Hanjoong Jo:** Conceptualization (equal); resources (equal); writing – review and editing (equal). **Joon Seon Song:** Data curation (equal); formal analysis (equal); resources (equal). **Soo‐jin Kang:** Conceptualization (equal); project administration (equal); supervision (equal); validation (equal); writing – original draft (equal). **Jun Ki Kim:** Conceptualization (equal); funding acquisition (equal); project administration (equal); resources (equal); supervision (equal); validation (equal); writing – original draft (equal); writing – review and editing (equal).

## CONFLICT OF INTEREST STATEMENT

Jun Ki Kim is a scientific advisor to the startup Apollon Inc. (Korea), which may develop diagnostic devices based on this work. The authors have declared that no other competing interests exist.

### PEER REVIEW

The peer review history for this article is available at https://www.webofscience.com/api/gateway/wos/peer‐review/10.1002/btm2.10529.

## Data Availability

The data has been included in the manuscript or Supporting Information. Additional generated or analyzed data are available from the corresponding author upon reasonable request.
